# The Interplay of UCP3 and PCSK1 Variants in Severe Obesity

**DOI:** 10.1007/s13679-025-00631-1

**Published:** 2025-04-26

**Authors:** Ludovica Verde, Martina Galasso, Dawn K. Coletta, Silvia Savastano, Lawrence J. Mandarino, Annamaria Colao, Luigi Barrea, Giovanna Muscogiuri

**Affiliations:** 1 Department of Public Health, University of Naples Federico II, Via Sergio Pansini 5, 80131 Naples, Italy; 2 Department of Medicine, Division of Endocrinology, University of Arizona, Tucson, AZ USA; 3 Dipartimento di Medicina Clinica e Chirurgia, Centro Italiano per la cura e il Benessere del Paziente con Obesità (C.I.B.O), Università degli Studi di Napoli Federico II, Via Sergio Pansini 5, 80131 Naples, Italy; 4 Department of Physiology, University of Arizona, Tucson, AZ USA; 5 Center for Disparities in Diabetes, Obesity and Metabolism, University of Arizona, Tucson, AZ USA; 6 Unità di Endocrinologia, Diabetologia e Andrologia, Dipartimento di Medicina Clinica e Chirurgia, Università degli Studi di Napoli Federico II, Via Sergio Pansini 5, 80131 Naples, Italy; 7 Cattedra Unesco “Educazione Alla Salute E Allo Sviluppo Sostenibile”, University Federico II, 80131 Naples, Italy; 8 Dipartimento di Psicologia e Scienze della Salute, , Università Telematica Pegaso, Centro Direzionale Isola F2, Via Porzio, Isola F2, 80143 Naples, Italy

## Abstract

**Summary:**

Obesity is a heterogeneous and multifactorial disease with a strong genetic component. While polygenic obesity accounts for most common cases, rare monogenic variants contribute, particularly in severe, early-onset obesity. Among the lesser-studied candidates are UCP3 and PCSK1, genes involved in key metabolic pathways.

**Recent Findings:**

The UCP3 p.Val192Ile (c.574G > A) and PCSK1 p.Asn221Asp (c.661 A > G) variants have been independently associated with metabolic pathways, including fatty acid oxidation and hormone processing, as well as a modestly increased risk of obesity. Clinical and genetic characterization of two patients with severe early-onset obesity revealed the co-occurrence of these variants, which were associated with metabolic disturbances such as insulin resistance.

**Purpose of the Review:**

This narrative review examined the functional and clinical significance of *UCP3* and *PCSK1* variants in severe obesity, presenting two case reports to illustrate their potential impact. Our findings support a potential model in which rare variants in distinct metabolic genes may interact synergistically to exacerbate disease severity. Further studies are needed to elucidate their combined functional effects and contributions to obesity pathogenesis.

## Introduction

Obesity is the most prevalent chronic disease in Western countries and a major risk factor for long-term metabolic complications [1, 2]. It is a heterogeneous and multifactorial condition, with heritability estimates ranging from 50 to 75%, particularly pronounced in severe, early-onset cases [1, 3]. Despite the well-established genetic component, the underlying causes of obesity remain incompletely understood, with a significant portion of its heritability still unexplained [1].

A minority of cases are syndromic, associated with distinctive clinical features and known genetic syndromes [4]. However, most individuals with obesity present with non-syndromic forms, in which highly penetrant rare variants—primarily inherited in an autosomal recessive manner—have been identified in a small subset (~ 2–5%) of patients. These variants affect key genes involved in energy balance, including leptin (LEP), LEP receptor (LEPR), melanocortin 4 receptor (MC4R), proprotein convertase subtilisin/kexin type 1 (PCSK1), pro-opiomelanocortin (POMC), melanocortin 3 receptor (MC3R), single-minded homolog 1 (SIM1), and neurotrophic receptor tyrosine kinase 2 (NTRK2) [4] (Table 1).

In contrast, the majority of obesity cases are believed to arise from a polygenic or multifactorial inheritance model, where common variants exert modest individual effects [4]. Genome-wide association studies (GWAS) have identified multiple loci associated with body mass index (BMI), such as fat mass and obesity-associated (FTO), uncoupling protein (UCP), brain-derived neurotrophic factor (BDNF), beta-3 adrenergic receptor (ADRB3), neuronal growth regulator 1 (NEGR1), proprotein convertase subtilisin/kexin type 2 (PCSK2), and peroxisome proliferator-activated receptor gamma (PPARG) (Table 2). Nonetheless, the strongest common variants account for only ~ 2% of BMI variance, indicating a limited explanatory power. Additionally, structural variants such as copy number variants (CNVs) contribute to the genetic architecture of obesity [4].

The identification of genetic factors underlying severe obesity is clinically relevant not only for personalized management and genetic counseling but also because emerging therapies specifically target genetically defined forms of obesity [5].

Although UCP3 and PCSK1 have been extensively studied, the phenotypic impact of their combined variants on obesity remains less explored [6, 7]. Both genes play crucial roles in metabolism and energy homeostasis, and variants affecting their function may contribute to the development of obesity [6, 7].

This review aimed to explore the emerging role of rare genetic variants in UCP3 and PCSK1 in non-syndromic obesity. We will summarize the current evidence linking these genes to obesity pathophysiology and present two novel clinical cases carrying both UCP3 p. Val192Ile (c.574G > A) and PCSK1 p.Asn221Asp (c.661 A > G) variants, contributing to the growing body of knowledge on the genetic underpinnings of obesity.

## Genetic Contributions to Obesity

### Monogenic Vs. Polygenic Obesity

Obesity can be broadly classified into monogenic and polygenic forms based on its genetic architecture [8]. Monogenic obesity refers to rare, highly penetrant cases caused by mutations in a single gene, often following autosomal recessive or dominant inheritance patterns. These cases typically present in early childhood with severe obesity and, in some instances, additional metabolic or neuroendocrine abnormalities. Key genes implicated in monogenic obesity include those involved in the leptin-melanocortin signaling pathway, such as MC4R, LEPR, PCSK1, and POMC, among others (Table 1) [8].

**Table 1:** Common genes associated with Monogenic obesity

Ref.	Gene	Function
[9]	Adenylate Cyclase 3 (ADCY3)	Disruption of primary cilia in neurons known to influence energy balance
[10]	Agouti Related Neuropeptide (AGRP)	Endogenous antagonist of MC4R, to which it binds to increase food intake
[11]	Brain-Derived Neurotrophic Factor (BDNF)	Supports survival of neurons; may influence eating behavior and energy balance
[12]	Kinase Suppressor of Ras 2 (KSR2)	Affects energy intake/expenditure and insulin sensitivity
[13]	Leptin (LEP)	Hormone secreted by adipose tissue that signals energy sufficiency
[13]	Leptin Receptor (LEPR)	Suppresses hunger by transmitting leptin’s signal to the brain
[14]	Melanocortin 4 Receptor (MC4R)	Influences appetite control via activation by α-MSH
[15]	Melanocortin 2 Receptor Accessory Protein 2 (MRAP2)	Enhances MC4R signaling and is essential for energy homeostasis
[16]	Neurotrophic Receptor Tyrosine Kinase 2 (NTRK2)	Receptor for BDNF; involved in energy balance and appetite regulation
[17]	Pleckstrin Homology Domain-Interacting Protein (PHIP)	Involved in insulin signaling and β-cell function
[18]	Pro-opiomelanocortin (POMC)	Precursor of α-MSH; contributes to reduced appetite via MC4R
[19]	Proprotein Convertase Subtilisin/Kexin Type 1 (PCSK1)	Involved in the maturation of insulin and other metabolic hormones
[20]	SH2B Adaptor Protein 1 (SH2B1)	A signaling molecule downstream of the leptin receptor
[21]	Single-minded Family BHLH Transcription Factor 1 (SIM1)	Regulates development of the paraventricular nucleus and expression of MC4R

MSH, melanocyte stimulating hormone

Mutations in these genes disrupt appetite regulation, energy expenditure, and neuroendocrine function, leading to early-onset, severe obesity [8].

In contrast, polygenic obesity results from the combined effect of multiple common genetic variants, each exerting a modest influence on body weight regulation [22, 23]. GWAS have identified over a hundred loci associated with BMI and obesity risk (Table 2) [22, 23].

**Table 2:** Common genes associated with polygenic obesity

Ref.	Gene (abbreviation)	Function
[24]	Amylase Alpha 1 A (AMY1A)	Produces salivary α-amylase, a key enzyme in starch digestion
[25]	Adrenergic Receptor Beta 3 (ADRB3)	Encode β-adrenergic receptors that regulate thermogenesis and lipolysis
[23]	Brain-Derived Neurotrophic Factor (BDNF)	Involved in neuronal development, energy balance, and appetite regulation
[26]	Cannabinoid Receptor 1 (CNR1)	Encodes the cannabinoid receptor 1; involved in appetite and energy balance
[27]	G Protein-Coupled Receptor Class C Group 5 Member B (GPRC5B)	May modulate insulin secretion
[28]	Melanocortin 4 Receptor (MC4R)	Governs appetite and autonomic responses by binding melanocortin peptides and AGRP
[29]	Neuronal Growth Regulator 1 (NEGR1)	Encodes a cell-adhesion molecule expressed in the brain
[30]	Proprotein Convertase Subtilisin/Kexin Type 1 (PCSK1)	Encodes a prohormone convertase involved in energy homeostasis and appetite
[31]	Peroxisome Proliferator-Activated Receptor Gamma (PPARG)	Regulates adipocyte differentiation and glucose metabolism
[32]	Pancreatic Polypeptide Receptor 1 (PPYR1)	Encodes a potent anti-obesity agent known to inhibit food intake
[33]	Uncoupling Protein 1–3 (UCP1-3)	Encode mitochondrial proteins that regulate energy expenditure and thermogenesis

AGRP, agouti-related protein

However, the effect sizes of these variants are small, and together they account for a small proportion of BMI heritability [22, 23].

Notably, certain genes such as MC4R, BDNF, and PCSK1 are implicated in both monogenic and polygenic obesity, depending on the type and frequency of the variant. Rare, highly penetrant mutations in these genes can cause severe early-onset obesity, while common variants are associated with modest increases in obesity risk in the general population.

### Key Metabolic Pathways Affected by Obesity Genes

Genes implicated in obesity predominantly affect metabolic pathways involved in appetite regulation, energy expenditure, and systemic metabolic homeostasis [1, 8]. Disruption of these pathways can lead to imbalances in energy intake and expenditure, ultimately contributing to excessive fat accumulation and obesity-related comorbidities [1, 8].

One of the most well-characterized pathways is the leptin-melanocortin signaling axis, which plays a central role in appetite regulation and energy balance [34]. Leptin, a hormone secreted by white adipocytes, binds to its receptor (LEPR) in the hypothalamus, initiating a signaling cascade that activates POMC neurons. These neurons produce α-MSH, which acts on the MC4R to suppress appetite and increase energy expenditure. Mutations in LEP, LEPR, POMC, and MC4R disrupt this regulatory loop, resulting in hyperphagia, reduced satiety, and early-onset obesity [34].

In addition to appetite control, genetic variants also influence energy expenditure and thermogenesis. Genes such as UCP3 modulate mitochondrial function and fatty acid oxidation, processes critical for adaptive thermogenesis and the regulation of lipid storage [35]. Variants in UCP3 that impair mitochondrial efficiency may lead to reduced energy expenditure and increased fat accumulation, thereby promoting an obese phenotype [35].

Furthermore, obesity-associated genes contribute to metabolic alterations, including altered glucose metabolism. For instance, mutations in PCSK1, which encodes the prohormone convertase 1, impair the processing of several prohormones [36]. In fact, PCSK1 is mainly expressed in neuroendocrine tissues, where it is involved in tissue-specific processing of prohormones and neuropeptide precursors such as proopiomelanocortin, proinsulin, proglucagon, and other known key regulators of energy metabolism [36]. This can result in a complex metabolic phenotype characterized by obesity, insulin-resistance, and other endocrine disturbances.

## UCP3 and its Role in Obesity

UCP3 is a member of the mitochondrial anion carrier protein family and is primarily predominantly expressed in skeletal muscle and brown adipose tissue, key sites of energy metabolism [37]. Located in the inner mitochondrial membrane, UCP3 plays a pivotal role in regulating energy homeostasis by modulating proton leak, thereby uncoupling oxidative phosphorylation from ATP synthesis. This uncoupling dissipates the proton gradient as heat, contributing to adaptive thermogenesis and the regulation of basal metabolic rate [37].

In addition to its thermogenic function, UCP3 is involved in fatty acid metabolism, particularly in the export of fatty acid anions and peroxides from mitochondria, a process essential for preventing lipotoxicity and maintaining mitochondrial function [38]. By facilitating fatty acid oxidation, UCP3 helps balance lipid utilization and storage, thereby influencing whole-body energy expenditure and metabolic flexibility [38].

Impaired function or reduced expression of UCP3 has been associated with decreased mitochondrial efficiency, impaired fatty acid oxidation, and increased triglyceride accumulation, particularly in skeletal muscle [39]. These alterations contribute to energy surplus, lipid storage, and insulin resistance, all of which are metabolic hallmarks of obesity [40]. Furthermore, genetic variants that reduce UCP3 activity may lead to diminished thermogenic responses and lower energy expenditure, predisposing individuals to positive energy balance and weight gain under conditions of caloric excess [37].

### UCP3 p.Val192Ile (c.574G > A): Evidence from Literature

At least 14 polymorphisms have been identified in the UCP3 gene and its promoter region, with functional analyses demonstrating that several of these variants lead to truncated proteins, increased expression, and reduced or absent protein activity [41]. However, association studies have yielded inconsistent results regarding their impact on obesity-related traits, with some demonstrating significant associations with BMI, body fat percentage, waist-to-hip ratio, skinfold thickness, respiratory quotient, and resting metabolic rate [42–46], while others reported no effects [47].

A study performed on 200 children with severe, early-onset obesity (BMI-SDS > 2.5; onset < 4 years) identified an association with the p.Val192Ile (c.574G > A) variant in three unrelated probands, all in the heterozygous state [35]. This missense mutation involves the substitution of valine with isoleucine—a non-polar, hydrophobic amino acid—at a highly conserved position within the fourth transmembrane domain of the II solcar repeat. Interestingly, functional assays in HEK293 cells demonstrated that this variant reduced fatty acid β-oxidation by 55%, with cells retaining only ~ 45% of the wild-type UCP3 activity, leading to increased triglyceride storage [35]. This impaired energy metabolism observed with the mutation may contribute to obesity development.

## PCSK1 and its Role in Obesity

PCSK1, also known as PC1/3, is a serine endoprotease that plays an essential role in the post-translational processing of prohormones into their biologically active forms [48]. It is predominantly expressed in neuroendocrine tissues, including the hypothalamus, pancreas, and intestinal enteroendocrine cells, where it facilitates the precise cleavage of prohormones and proneuropeptides at specific recognition sites [49].

PCSK1 is critically involved in the processing of key metabolic hormones, including proinsulin to insulin, POMC to melanocyte-stimulating hormones (e.g., α-MSH), and proglucagon to GLP-1 [48]. Through these actions, PCSK1 directly influences glucose metabolism, insulin sensitivity, and appetite regulation. Defective PCSK1 activity leads to incomplete or inefficient hormone processing, resulting in hyperproinsulinemia, impaired insulin secretion, and dysregulation of satiety signals, all of which are linked to insulin resistance, hyperglycemia, and increased food intake [48].

Mutations in PCSK1 have been associated with monogenic forms of obesity and endocrine disorders, including congenital proprotein convertase 1 deficiency, characterized by early-onset obesity, hypoglycemia, and malabsorptive diarrhea [50]. Even heterozygous missense variants with partial loss of function may contribute to polygenic obesity through subtle but chronic disruptions in metabolic and appetite-regulating pathways [50].

### PCSK1 p.Asn221Asp (c.661 A > G): Evidence from Literature

To assess the contribution of PCSK1, also known as PC1/3, to polygenic obesity risk, a large study genotyped tag SNPs in 13,659 individuals of European ancestry across eight independent cohorts. The nonsynonymous variant rs6232, encoding Asn221Asp, showed a significant association with obesity in both adults and children (*P* = 7.27 × 10⁻⁸) [51]. Functional analyses demonstrated a significant reduction (10.4%; *P* = 0.03) in catalytic activity of the Asn221Asp-mutant PC1/3 protein compared to the wild-type enzyme, although no alteration in maturation or secretion was observed in lysate or medium [51].

The Asn221Asp substitution occurs in the catalytic domain of PC1/3 and is located in close proximity to two other polymorphic sites—Glu250stop and Ala213del—which lead to protein truncation and deletion of a conserved alanine residue near the catalytically critical His208 [52]. Importantly, Asn221Asp has direct contact with His208, suggesting a possible mechanistic basis for the observed reduction in enzymatic activity [51]. Furthermore, Asn221Asp is adjacent to Asn222Asp, a murine PC1/3 variant that causes maturity-onset obesity and increased body fat in homozygous mice; heterozygous mice also exhibit higher fat content compared to wild-type controls [53].

For comparison, the Q665E-S690T variant cluster, also associated with obesity (*P* = 2.31 × 10⁻¹²), did not significantly affect PC1/3 activity or processing, while G593R, known to cause human PC1/3 deficiency, led to a drastic 98.7% reduction in catalytic activity and altered protein maturation [48, 51]. Overall, these findings suggest a modest but functionally relevant impairment in PC1/3 activity caused by Asn221Asp, which may contribute to the pathogenesis of obesity [51]. Additional research into noncatalytic roles of PC1/3 and regulatory variants in high linkage disequilibrium (LD) with rs6232 may further clarify their collective impact on obesity susceptibility. Finally, in terms of clinical significance, the PCSK1 p.Asn221Asp variant has been associated with BMI quantitative trait locus 12, highlighting its potential contribution to obesity risk [22].

## Case Reports

**Patient 1** was a 37-year-old woman seeking medical and surgical weight loss options after unsuccessful attempts at weight loss through lifestyle changes. Medical history was significant for hypothyroidism, hyperuricemia, and vitamin D deficiency. At her initial visit, this patient actively took levothyroxine for hypothyroidism and allopurinol for hyperuricemia, and we prescribed calcifediol for vitamin D deficiency. She also reported a history of pediatric obesity. At her initial appointments, this patient weighed 146 kg (BMI: 57 kg/m²). Physical exam demonstrated excess abdominal adiposity. The body composition assessed by bioimpedance was the following: R (W) Initial laboratory evaluation was significant for insulin resistance (HOMA-IR: 3.1). Workup for potential secondary causes of obesity was normal. Due to her lack of weight loss, history of pediatric obesity, and high suspicion of genetic contribution to weight gain, genetic testing was performed, and the variant c.574G > A p.(Val192Ile) and the variant c.661 A > G p.(Asn221Asp) were detected, both in heterozygosity, in the UCP3 and PCSK1 genes, respectively.

**Patient 2** was a 56-year-old man referred to our obesity outpatient clinic for weight loss. He reported to be affected by hypothyroidism and vitamin D deficiency. At his initial visit, this patient reported taking levothyroxine for hypothyroidism. He reported being affected by obesity since he was a child. Excess abdominal adiposity was detected at physical exam. At his initial appointments, his weight was 111 kg (BMI: 39 kg/m²). Physical exam demonstrated excess abdominal adiposity. Laboratory evaluation detected the presence of insulin resistance (HOMA-IR: 5.8) and hyperuricemia. Based on the history of pediatric obesity and high suspicion of genetic contribution to weight gain, genetic testing was performed, and the variant c.574G > A p.(Val192Ile) and the variant c.661 A > G p.(Asn221Asp) were detected, both in heterozygosity, in the UCP3 and PCSK1 genes, respectively.

## Synergistic Interactions Hypothesis

**Fig. 1:**
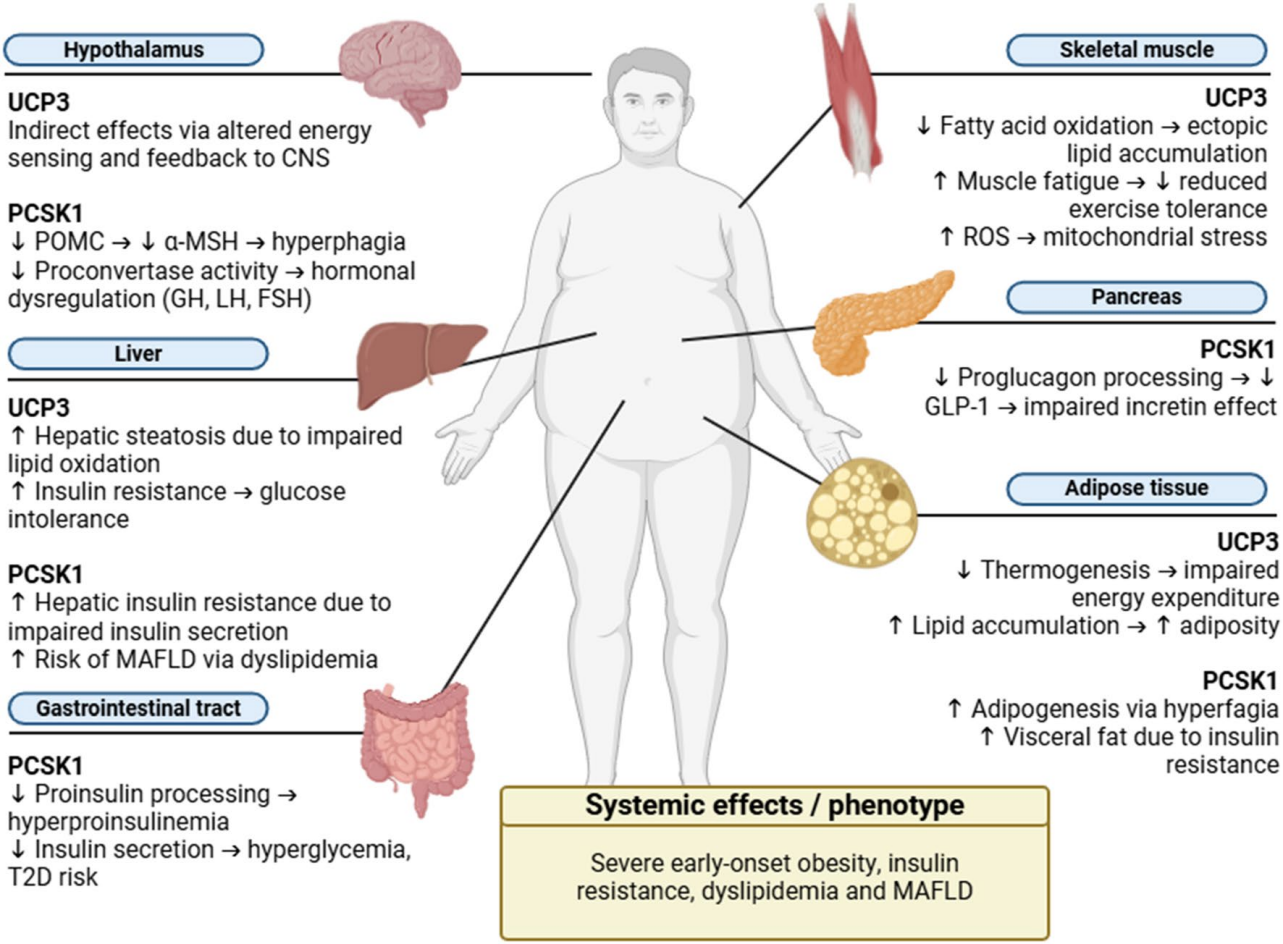
Synergistic impact of UCP3 and PCSK1 variants on metabolic pathways driving severe early-onset obesity. Schematic representation of the metabolic consequences associated with pathogenic variants in UCP3 and PCSK1, highlighting their combined contribution to a distinct severe early-onset obesity phenotype. UCP3 dysfunction impairs fatty acid oxidation, thermogenesis, and energy expenditure, particularly affecting skeletal muscle, adipose tissue, and liver, with downstream effects on insulin sensitivity. PCSK1 variants disrupt prohormone processing (e.g., POMC, proinsulin, and proglucagon), leading to hyperphagia, insulin resistance, and glucose dysregulation, impacting the neuroendocrine axis, pancreas, liver, and gastrointestinal tract. The synergistic effect of these genetic alterations results in pronounced adiposity, dyslipidemia, and increased risk of type 2 diabetes (T2D) and metabolic-associated fatty liver disease (MAFLD). Abbreviations: GLP-1, Glucagon-Like Peptide-1; POMC, Proopiomelanocortin; α-MSH, Melanocyte Stimulating Hormone; GH, Growth Hormone; LH, Luteinizing Hormone; FSH, Follicle-Stimulating Hormone; ROS, Reactive Oxygen Species; MAFLD, Metabolic Dysfunction-Associated Fatty Liver Disease; T2D, Type 2 Diabetes; CNS, Central Nervous System

The co-occurrence of UCP3 c.574G > A p.(Val192Ile) and PCSK1 c.661 A > G p.(Asn221Asp) variants in both patients raises the possibility of an additive effect on metabolic dysregulation (Fig. 1). As described above, each variant individually has been shown to impair metabolism pathways; however, their simultaneous presence, as described in the case reports, suggests that these mutations may amplify across several metabolic pathways. For example, UCP3 variants affect fatty acid oxidation and energy expenditure [6, 37, 43], while PCSK1 mutations impact neuroendocrine signaling and appetite regulation [36, 51]. Taken together, these variants may promote a more severe obese phenotype than mutation alone. Although direct functional validation of their combined impact is lacking, this underscores the need for future studies exploring the cumulative burden of rare variants in energy balance pathways.

## Broader Implications and Future Perspectives

The identification of rare genetic variants associated with obesity underscores the importance of genetic testing, particularly in individuals with severe, early-onset obesity or family histories suggestive of heritable metabolic disorders [54]. Genetic analysis can provide diagnostic clarity, inform risk stratification, and guide personalized clinical management, including nutritional, pharmacological, and behavioral interventions tailored to the individual’s genetic profile [54].

Advancements in NGS have facilitated the discovery of rare, potentially pathogenic variants in genes involved in energy metabolism, offering new insights into the molecular mechanisms underlying obesity [55, 56]. These findings hold promise for personalized medicine, where targeted therapies—such as agents that enhance fatty acid oxidation or improve prohormone processing—may be developed to address specific metabolic defects [55, 56].

However, functional validation of rare variants remains a critical challenge [57–59]. In vitro and in vivo studies are needed to elucidate the biological impact of these mutations and to determine their causal role in obesity pathogenesis. Such studies are essential for distinguishing pathogenic variants from benign polymorphisms, thereby improving the interpretation of genetic findings in clinical settings [57–59].

Furthermore, the exploration of novel therapeutic targets based on gene function, for instance, enhancing UCP3-mediated thermogenesis or PCSK1-dependent hormone maturation, may open new avenues for precision interventions [55, 56]. Ultimately, integrating genetic, clinical, and metabolic data will be key to advancing personalized treatment strategies and improving outcomes for patients with genetically influenced forms of obesity [55, 56].

## Conclusion

Our review highlights the potential clinical relevance of rare, functionally significant variants in UCP3 and PCSK1 in patients with severe obesity. While each gene plays a distinct role in energy metabolism and endocrine regulation, their combined disruption may amplify metabolic dysfunction and contribute to a more severe obese phenotype. Our findings emphasize the importance of integrating genetic testing, especially in patients with early-onset disease or strong personal/family histories suggestive of genetic predisposition. We recommend that functional validation and exploring variant-variant interactions within relevant genes such as UCP3 and PCSK1 will be critical to improving our understanding of monogenic (rare) obesity and refining personalized treatment strategies.

## Data Availability

No datasets were generated or analysed during the current study.
